# Supplementary material for: Comparison Approach for Identifying Missed Invasive Fungal Infections in Formalin-Fixed, Paraffin-Embedded Autopsy Specimens
Source: J Fungi (Basel). 2022 Mar 24;8(4):337. doi: 10.3390/jof8040337 (PMC9030445; doi:10.3390/jof8040337)
Supplement: Supplementary file 1 [file jof-08-00337-s001.zip › jof-1645969-supplementary.pdf]

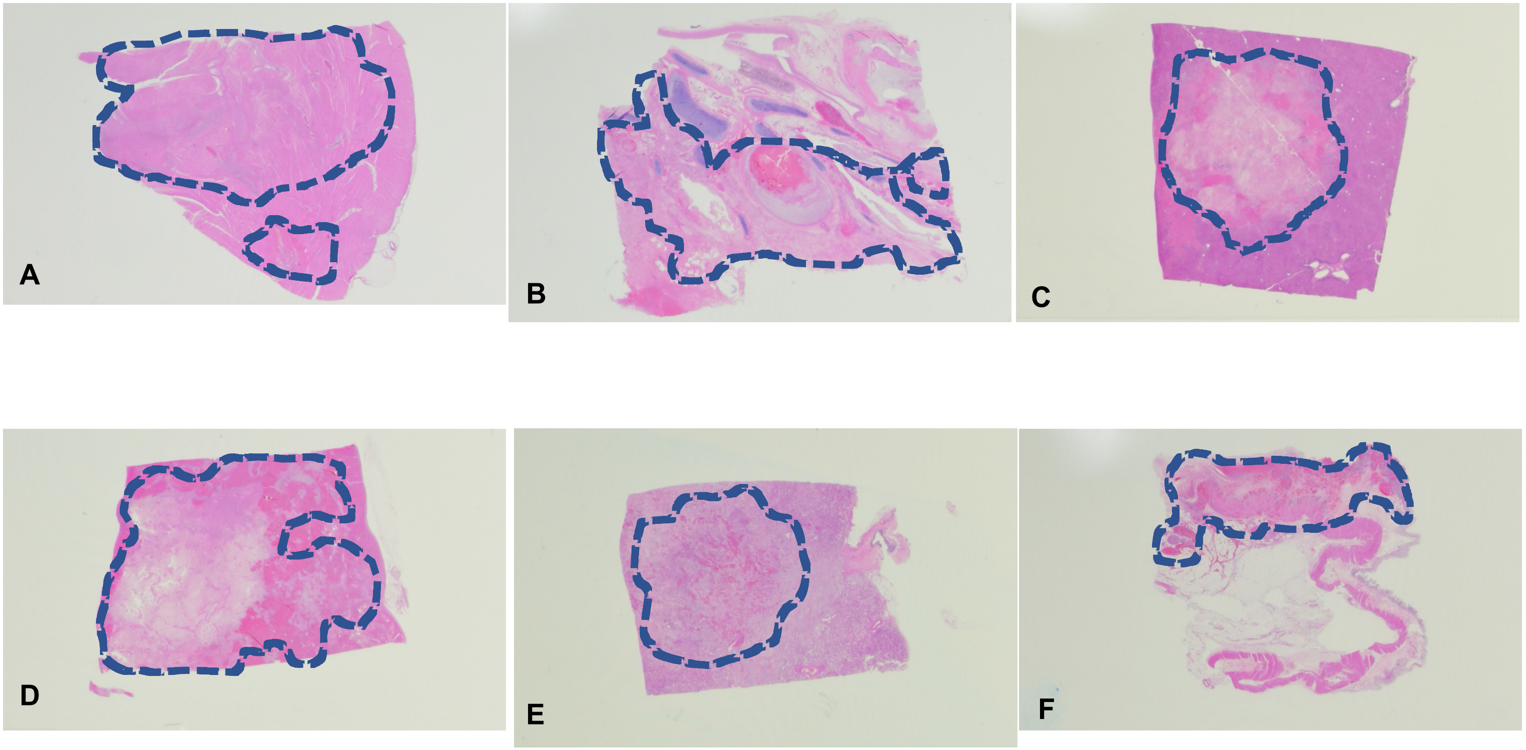

Figure S1. The area surrounded by the dotted line shows the fungal elements in each organ FFPE specimen. Macroscopic picture of hematoxylin and eosin slides. (A) Heart, 70%. (B) Lung, 60%. (C) Liver, 50%. (D) Spleen, 80%. (E) Kidney, 50%. (F) Colon, 40%.

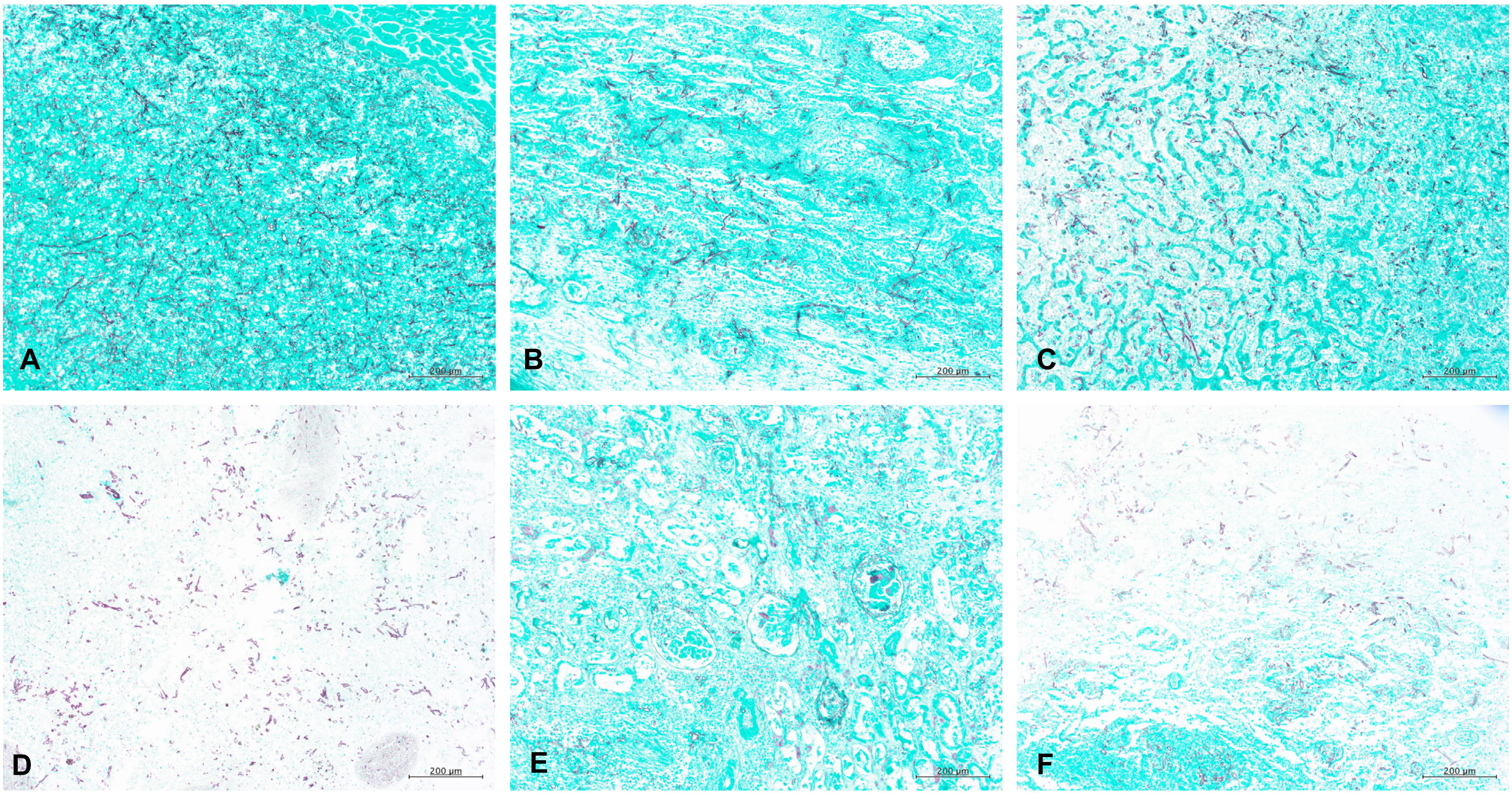

Figure S2. The density of fungal components in each formalin-fixed, paraffin-embedded specimen in each organ. Grocott's stain (100×). The density of fungi was classified as low, medium, or high. (A) Heart, high density. (B) Lung, medium density. (C) Liver, low density. (D) Spleen, medium density. (E) Kidney, low density. (F) Colon, low density.

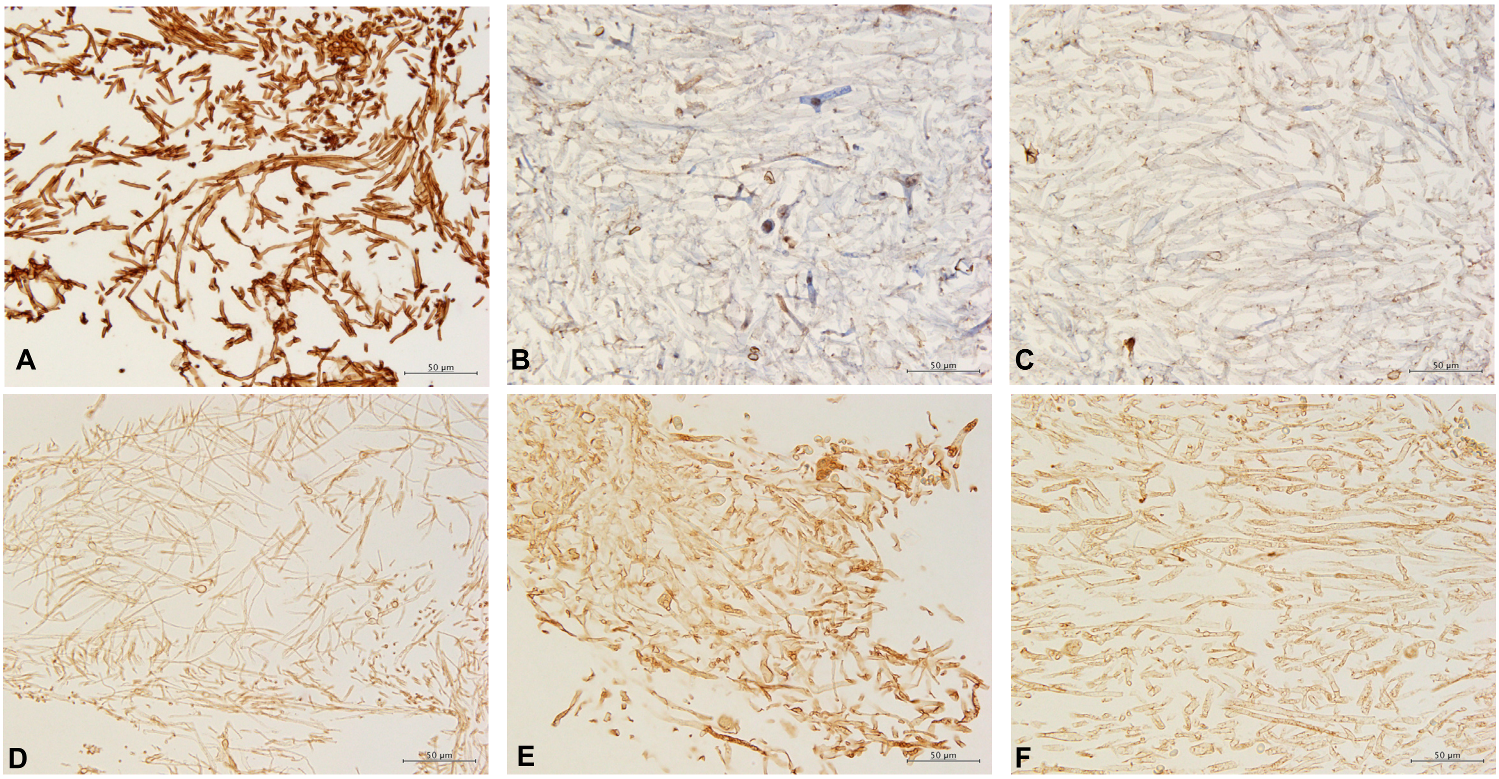

Figure S3. Immunohistochemistry (IHC) result of cultured fungi. The cultured fungi are as follws: (A) and (D), *Aspergillus fumigatus*; (B) and (E), *Rhizopus oryzae*; (C) and (F), *Cunninghamella elegans.*

Anti-*Aspergillus* antibody tested (A), (B) and (C), Anti-*Rhizopus arrhizus* antibody tested (D),(E) and (F). Magnification of all pictures is (400×).

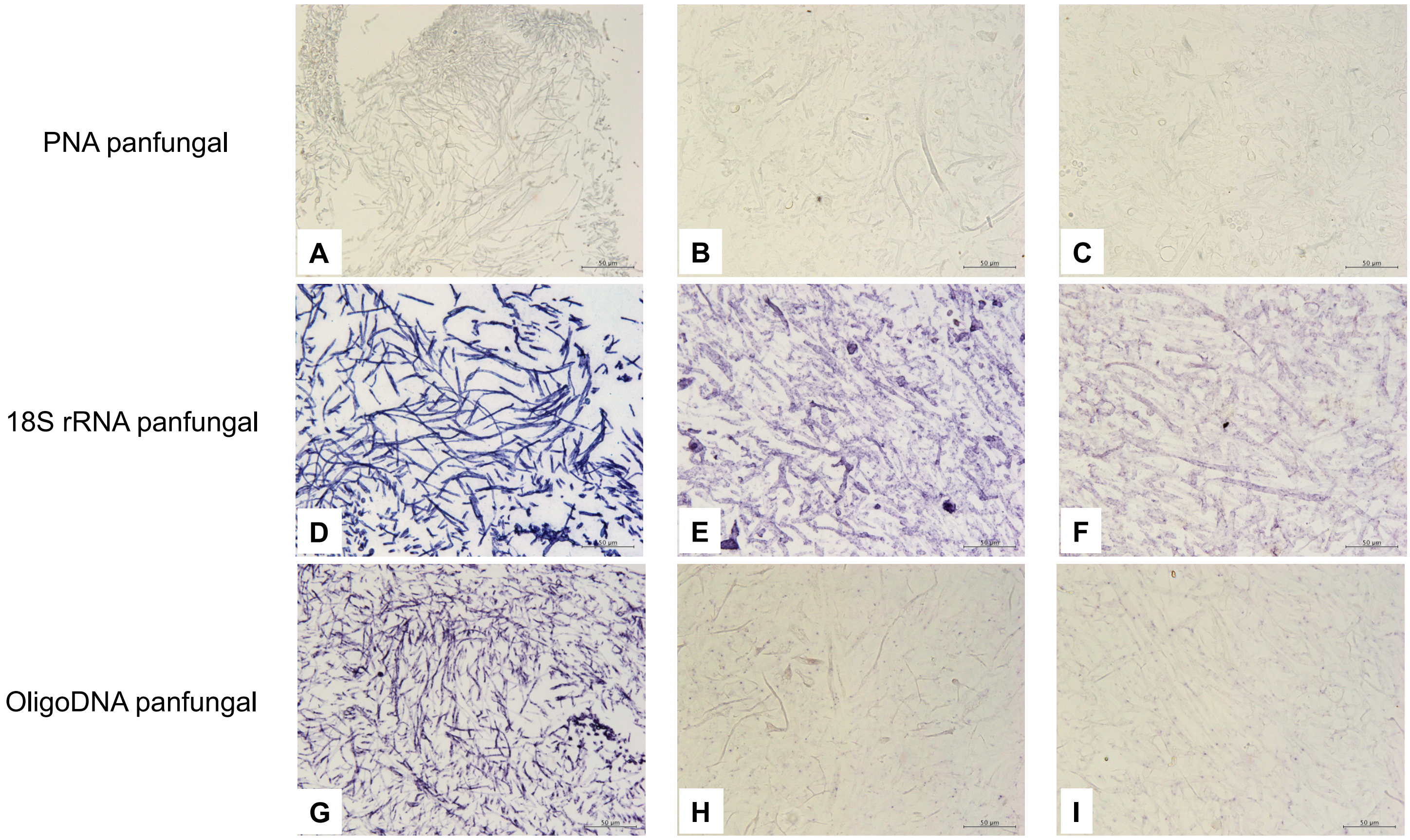

Figure S4. *In situ* hybridization (ISH) result of panfungal probes applied to cultured fungi. The cultured fungi are as follws: *Aspergillus fumigatus,* (A), (D) and (G); *Rhizopus oryzae,* (B), (E) and (H); *Cunninghamella elegans,* (C), (F) and (I). Probe tested are as follows: PNA panfungal probe, (A), (B) and (C); 18S rRNA panfungal probe, (D), (E) and (F); OligoDNA panfungal probe, (G), (H) and (I). Magnification of all pictures is (400×).

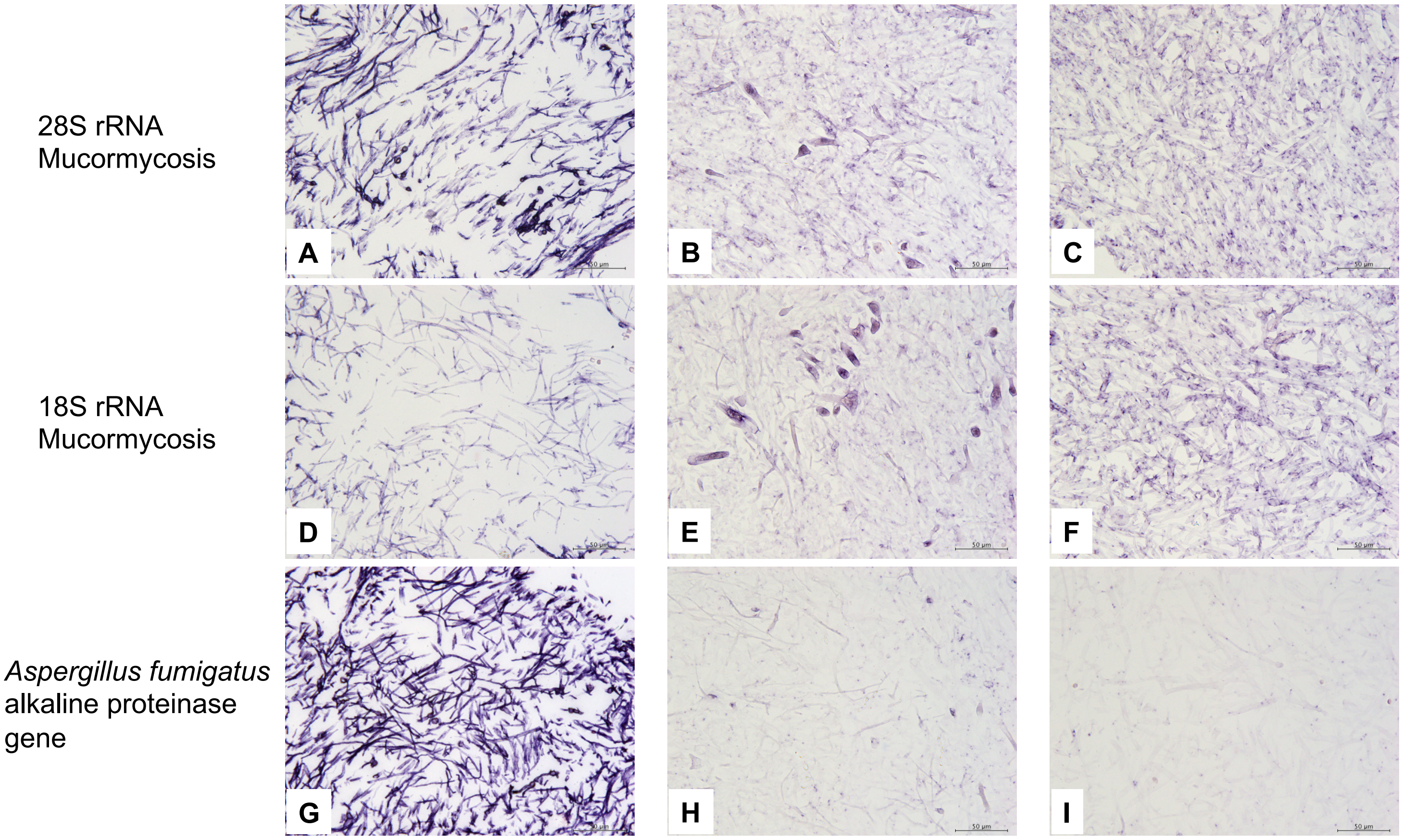

Figure S5. *In situ* hybridization (ISH) result of species-specific probes applied to cultured fungi. The cultured fungi are as follws: *Aspergillus fumigatus,* (A), (D) and (G); *Rhizopus oryzae,* (B), (E) and (H); *Cunninghamella elegans,* (C), (F) and (I). Probe tested are as follows: 28SrRNA Mucormycosis probe, (A), (B) and (C); 18SrRNA Mucormycosis probe, (D), (E) and (F); *Aspergillus fumigatus* alkaline proteinase gene probe, (G), (H) and (I). Magnification of all pictures is (400×).

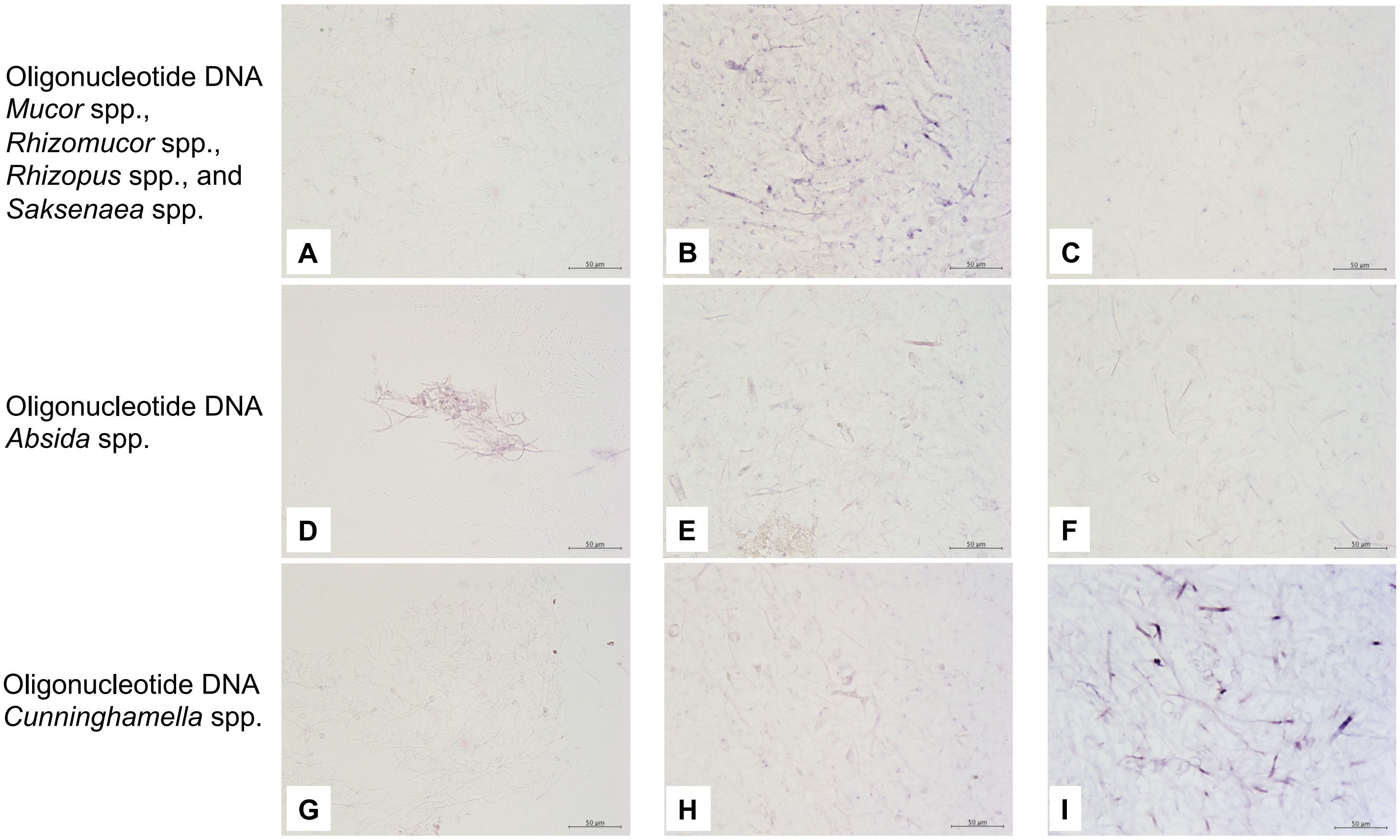

Figure S6. *In situ* hybridization (ISH) result of species-specific oligonucleotide probes applied to cultured fungi. The cultured fungi are as follws: *Aspergillus fumigatus,* (A), (D) and (G); *Rhizopus oryzae,* (B), (E) and (H); *Cunninghamella elegans,* (C), (F) and (I). Oligonucleotide probe tested are as follows: *Mucor* spp., *Rhizomucor* spp., *Rhizopus* spp., and *Saksenaea* spp. probe, (A), (B) and (C); *Absida* spp. probe, (D), (E) and (F); *Cunninghamella* spp. probe, (G), (H) and (I). Magnification of all pictures is (400×).

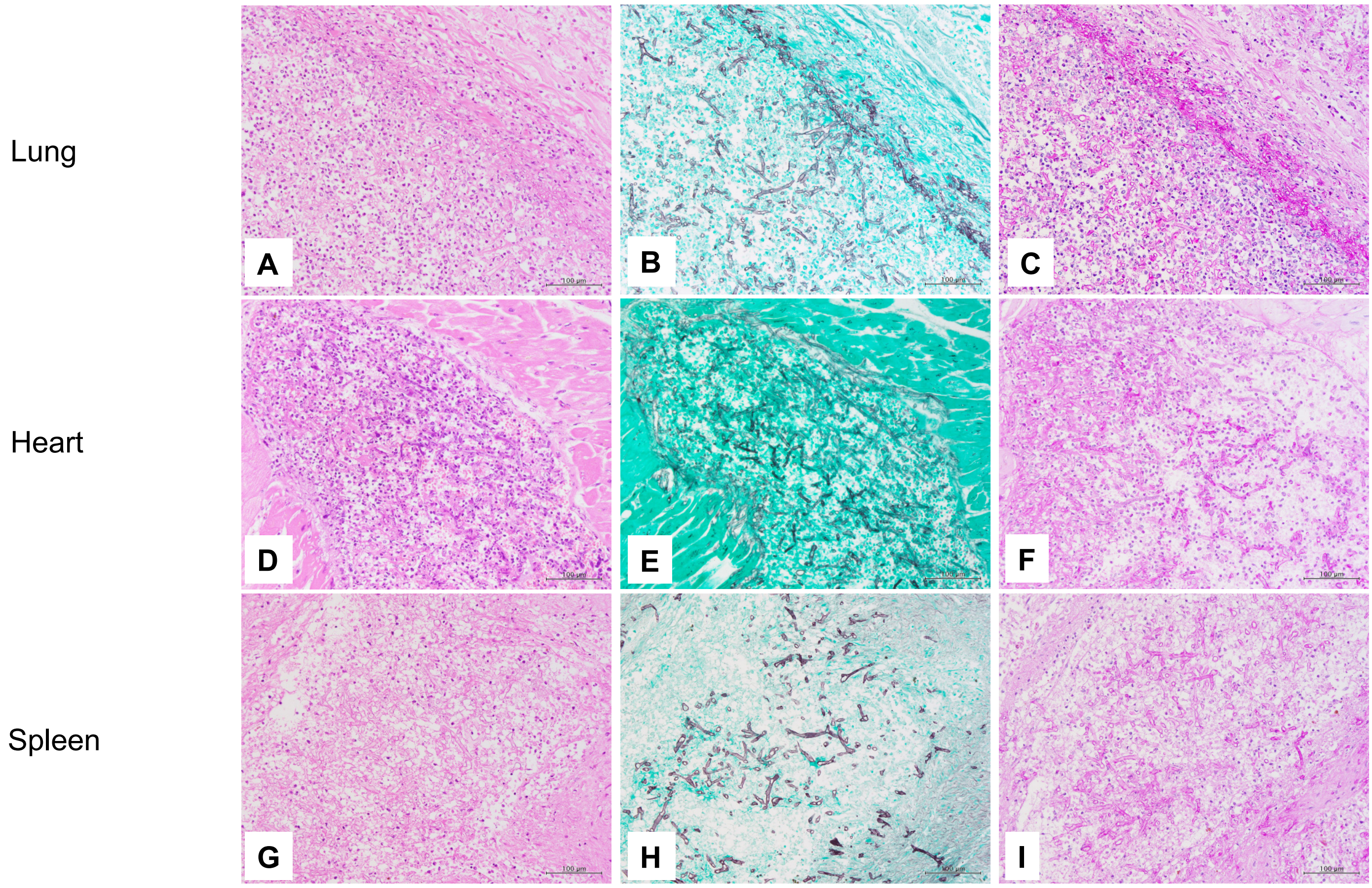

Figure S7. Summary of histopathology findings. Pictures (A), (B), and (C) are taken from the same area of the lung. Pictures (D), (E), and (F) are taken from the same site of the heart. Picture present in (G), (H), and (I) are taken from the same area of the spleen. Figure 2 is identical to Figure S7 (B). Figure 3 (A-B, E-F) are same as Figure S7 (D-E, G-H)

Staining is as follows: Hematoxylin eosin (HE) staining, (A), (D) and (G); Grocott's methenamine silver (GMS) staining, (B), (E) and (H); Periodic acid-Schiff (PAS) staining, (C), (F) and (I). Magnification of all pictures is (200×).
